# Personalized Hip Joint Replacement with Large Diameter Head: Current Concepts

**DOI:** 10.3390/jcm11071918

**Published:** 2022-03-30

**Authors:** Pascal-André Vendittoli, Sagi Martinov, Mina Wahba Morcos, Sivan Sivaloganathan, William G. Blakeney

**Affiliations:** 1 Surgery Department, Hôpital Maisonneuve-Rosemont, Montreal University, 5415 Boulevard de l’Assomption, Montreal, QC H1T 2M4, Canada; sagimartinov@gmail.com (S.M.); mina.wahbamorcos@mail.mcgill.ca (M.W.M.); sivan_shankar@hotmail.co.uk (S.S.); 2 Clinique Orthopédique Duval, Laval, QC H7M 2Y3, Canada; 3 Personalized Arthroplasty Society, Atlanta, GA 30305, USA; 4 Department of Orthopedic Surgery, Royal Perth Hospital, Perth, WA 6000, Australia; blakeney@gmail.com

**Keywords:** hip, total hip arthroplasty, personalized, personalised, anatomical, large diameter head, hip resurfacing, ceramic, dual mobility, enhanced recovery after surgery, offset, leg length, forgotten joint

## Abstract

Hip arthroplasty is a common procedure in elective orthopaedic surgery that has excellent outcomes. Hip replacement surgery aims to create a “forgotten” joint, i.e., a pain-free joint akin to a native articulation. To achieve such goals, hip arthroplasty must be personalised. This is achieved by restoring: the centre of rotation of the native hip; leg length equality; femoral offset; femoral orientation; soft tissue tension; joint stability with an unrestricted hip range of motion; and having appropriate stress transfer to the bone. In addition, the whole pathway should provide an uneventful and swift postoperative recovery and lifetime implant survivorship with unrestricted activities. At our institution, the preferred option is a personalized total hip arthroplasty (THA) with a large diameter head (LDH) using either monobloc or dual-mobility configuration for the acetabular component. LDH THA offers an impingement-free range of motion and a reduced risk of dislocation. The larger head-neck offset allows for a supraphysiologic range of motion (ROM). This can compensate for a patient’s abnormal spinopelvic mobility and surgical imprecision. Additionally, LDH bearing with a small clearance exerts a high suction force, which provides greater hip micro-stability. With appropriate biomechanical reconstruction, LDH THA can restore normal gait parameters. This results in unrestricted activities and higher patient satisfaction scores. We use LDH ceramic on ceramic for our patients with a life expectancy of more than 20 years and use LDH dual mobility bearings for all others.

## 1. Introduction

Hip replacement surgery has significantly evolved since its conception. Sir John Charnley, in his initial patient selection for the procedure, was looking ‘…for factors which offer a “built-in restraint”, such as defective knees or ankles, and impose some general physical limitations on the patient… to hold back physical activity….’ Despite the pioneers’ initial failures, it has gained momentum, with ever improving implant survivorship and patient satisfaction. Modern hip arthroplasty has shown outstanding results, and it was even named “the operation of the century” [1]. Nevertheless, the era of a standardized procedure for all patients is being superseded by the recent movement to create a more bespoke approach to the native anatomy. The ultimate aim of a hip replacement is to create a pain-free joint with the same functional movements as the native joint; this was described by Behrand et al. as the “forgotten” joint [2].

Hence, today the primary goal of THA is to recreate a pain-free native hip perception and function. Patients want to resume the same leisure activities as before the hip disease process (for example, sports) and return to work promptly after surgery. Some patients physically perform demanding jobs, such as roofing, plumbing, firefighter, police officer, etc., and not being able to resume their work and reorient their career can be problematic. Finally, patients naturally hope to undergo complication-free surgery with minimal postoperative pain and a swift recovery.

Ferguson et al. reported the most common causes of THA revision are aseptic loosening (48%), dislocation (15%), periprosthetic fracture (10%), infection (9%), and implant malpositioning (5%) [3]. The growing number of young and active patients undergoing THA, as well as the globally ageing population, are the leading causes for the increase in the complication rate, especially with regard to periprosthetic fractures and infections [4,5]. Some complications that require revision, such as a traumatic periprosthetic fracture or late infection, are difficult to prevent by improving the implant design or using new technology. The other significant causes for revision, however, such as dislocation or implant malpositioning, can be significantly minimized by improving hip biomechanics with personalized implant reconstruction [3]. 

## 2. What Is a Personalized/Optimal Hip Arthroplasty?

There is anatomic variation in the hip across individuals. The precise restoration of this anatomy during THA may improve clinical function and patient satisfaction. The improvement in the wear resistance of implants, fixation methods, and the unprecedented advancement in navigation and robotic technology affords a more precise anatomical hip restoration [6]. Additionally, patients may have specific medical or psychological conditions which need to be considered in order to optimize their outcomes. Patients’ activities of daily living, leisure, and related expectations also play an essential role in evaluating a successful THA. A personalized hip arthroplasty should aim to restore/provide:

1- Functional biomechanics;
  (a) The native hip’s centre of rotation;
  (b) Leg length equality;
  (c) Femoral offset and abductor lever arm;
  (d) Femoral orientation: Neck-shaft angle and version;
  (e) Balanced soft tissue tension;
2- Appropriate stress transfer from implant to bone (minimizing problematic bone remodeling, osteopenia, and thigh pain);
3- Hip range of motion and stability;
  (a) Stable joint;
  (b) Micro stability;
  (c) Impingement free ROM;
4- A forgotten joint;
5- Bearing wear resistance provides a lifetime implant survivorship with unrestricted activities;
6- Rapid and complication-free recovery.

**Figure 1:**
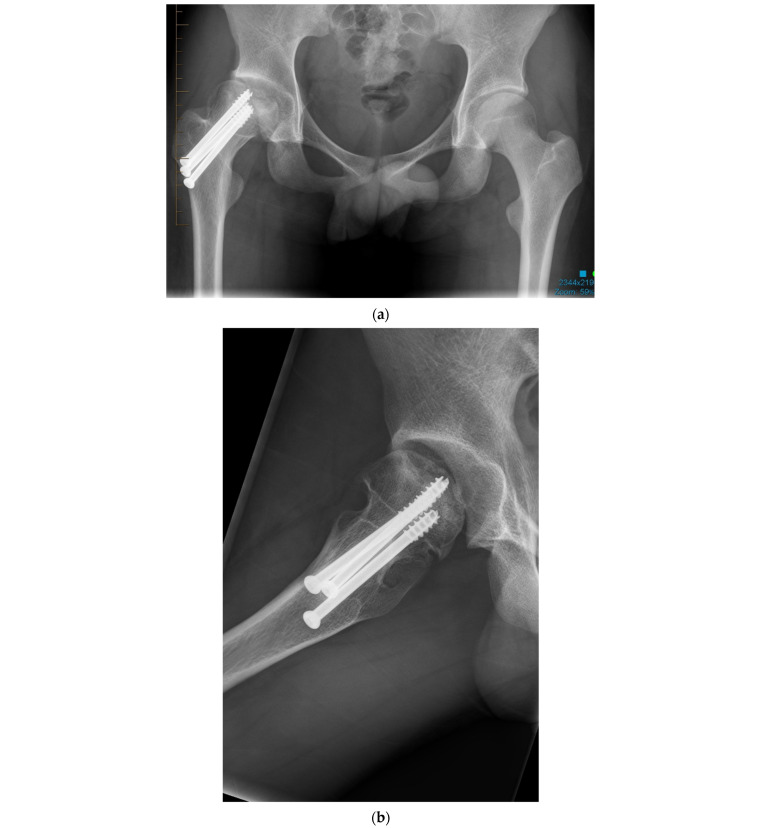

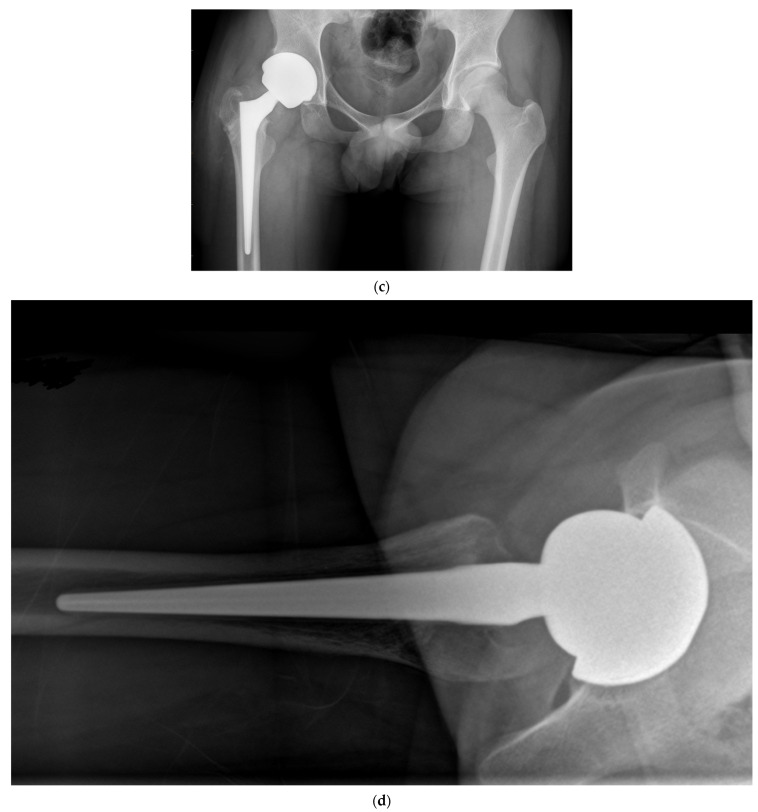
(**a**) Anteroposterior pelvis radiograph of 19-year-old men with right femoral head avascular necrosis following a slipped capital femoral epiphysis treated with canulated screws. During preoperative templating, acetabular, and femoral components sizes were estimated to be small. (**b**) Preoperative lateral view of the right hip. (**c**) Postoperative anteroposterior pelvis radiograph showing a CoC LDH THA with a 13 mm conical stem and a 46 mm monobloc ceramic acetabular component with a bearing diameter of 36 mm. (**d**) Postoperative lateral view of the right hip replacement.

This paper will discuss the advantages of a large diameter head (LDH) THA. An LDH bearing is defined as a femoral head >36 mm and/or a cup-head diameters difference of 12 mm, i.e., 46 mm cup with a 36 mm head (Figure 1). LDH THA and hip resurfacing became widely accepted in the 1990s. The popular bearing at the time was Metal on Metal (MoM). This bearing delivered a solution to the problem of early wear and failure of metal-polyethylene (MoP) bearings in younger, more active individuals. The LDH also provided superior functional ability and stability compared to their conventional standard diameter head THAs. As complications associated with MoM began to rise, other bearing options, such as Ceramic on Ceramic (CoC), for LDH became more popular. 

LDH THA is mainly available in two designs: Ceramic on Ceramic LDH THA with a monobloc acetabular cup or dual mobility (DM) LDH THA.

## 3. Two Types of Large Diameter Head THA Are Reviewed in This Article

### 3.1. CoC LDH THA

**Figure 2:**
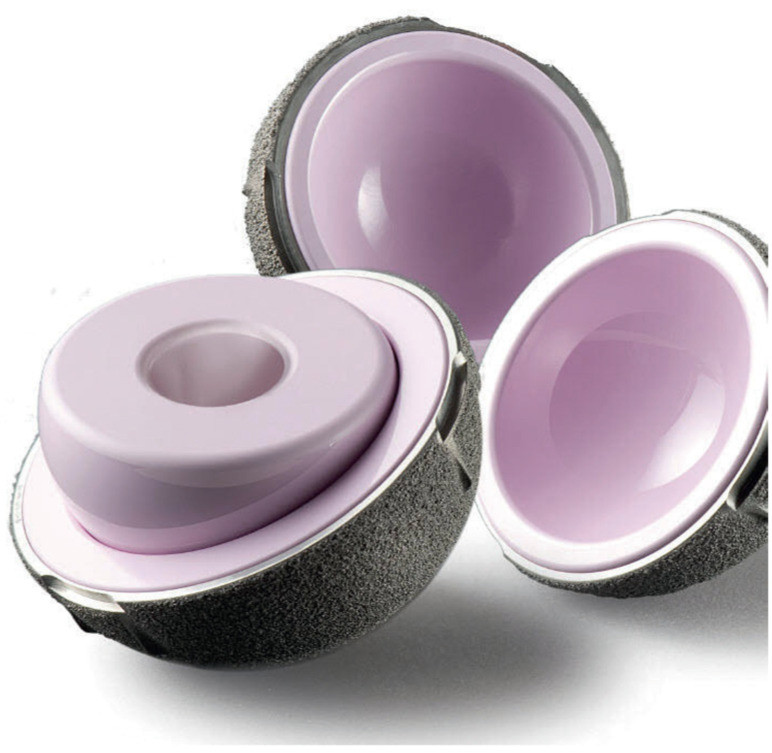
LDH ceramic monobloc acetabular component and ceramic head.

The use of CoC bearings, based on data from a number of national arthroplasty registers in 2018, is the third most widely selected bearing surface globally [7]. It has demonstrated a higher wear resistance and biocompatibility when compared to other materials [8]. However, LDH CoC THAs require a thin metallic acetabular shell to optimize the ratio of the bearing diameter to cup diameter. To reduce the risk of liner fracture due to incorrect liner assembly in a deformed metallic shell, CoC LDH cups are manufactured pre-assembled and then implanted as a monoblock component. Our practice has been using LDH CoC since 2011 and has implanted more than 3500 Maxera bearings (Zimmer, Warsaw, IN, USA, Figure 2). This shell is made of Titanium (Ti-6Al-4V) alloy with a thin layer of titanium vacuum plasma coating (Ti-VPS) on the shell’s exterior. The BIOLOX delta taper liner is pre-assembled and secured into the shell using an 18° taper angle. This implant has outer diameters ranging from 42 to 66 mm and articulation diameters ranging from 32 to 48 mm.

### 3.2. Dual Mobility LDH THA

**Figure 3:**
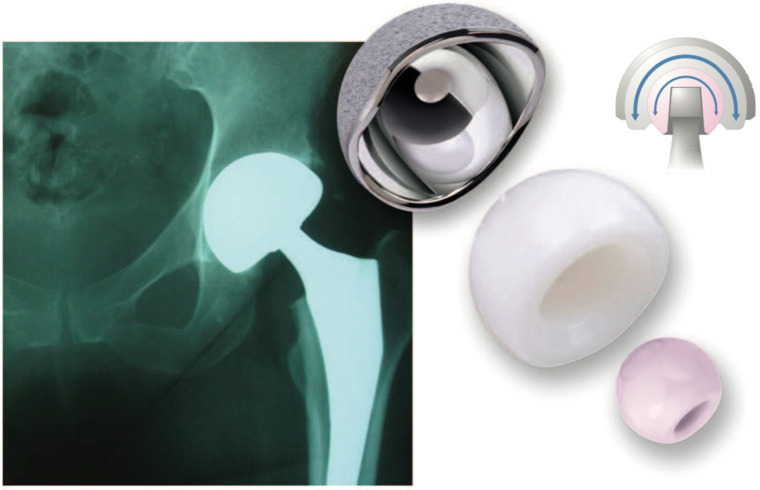
Composite image showing a monobloc DM acetabular component and its two main articulations: large polyethylene ball articulating with the metallic cup and the smaller articulation between the 28 mm ceramic head and the polyethene head (mobile liner).

In the 1970s, Professor Giles Bousquet, introduced the DM design. It incorporated both Charnley’s ‘low friction’ and McKnee–Farrar’s stability through the use large head principles [9,10]. DM design consists of a small articulation between the small femoral head (22 or 28 mm) captive and mobile within a polyethylene (PE) Liner (Figure 3) and a larger articulation between the PE liner ball with a highly polished metallic acetabular shell. The PE large head diameter is usually 6–8 mm smaller than the size of the outer metallic shell. Most of the movement occurs at the small articulation. Movement of the large articulation only happens when the stem’s neck comes into contact with the PE head. This design has two articulations (femoral head-PE liner, PE liner–outer shell) and three interfaces (small and larger bearing and neck-polyethylene contact point). Wear can occur at the two bearing surfaces and the taper junction. Particles produced (PE or metal) are dependent on the surfaces interacting.

#### 3.2.1. Goal #1 of a Personalized THA: Restore Hip Functional Biomechanics

(1a) The native hip’s centre of rotation

There are several benefits to restoring the native hip’s centre of rotation, all of which can result in a better patient outcome score. These benefits can include: a reduction in the risk of impingement (allowing for a greater range of movement and a decreased risk of dislocation); improved abductor function and hip kinematics; decreased implant wear; and a reduced long-term risk of implant loosening [11]. In most patients, the native acetabulum is sub-hemispherical, but the acetabular components used for THA are hemispherical. The mean subtending angle of the native acetabulum varies from 145° to 173° [12,13]. As an example, the height difference between a 58 mm/180° cup over a 58 mm/165° acetabulum is 2.4 mm [14]. This results in a lateral displacement of the centre of rotation when the implant is fully contained. Therefore, to restore the hip centre of rotation with a non-anatomical 180° cup, the surgeon must deepen (ream more medially) the native acetabulum. However, the centre of rotation of the prosthetic hip joint depends not only on the depth of reaming but also depends on the cup design (the polar/apex thickness of the cup and the liner). For example, 180° cup with uniform wall thickness requires less medial reaming than a cup with a lateralized centre of rotation (i.e., greater polar thickness) to achieve the same optimal centre of rotation. With a more anatomical acetabular component (165°), the hip’s anatomical center of rotation could be restored more precisely with less aggressive reaming [12].

(1b) Leg length equality,
(1c) femoral offset and abductors lever arm,
(1d) femoral orientation,
(1e) soft tissue tension

**Figure 4:**
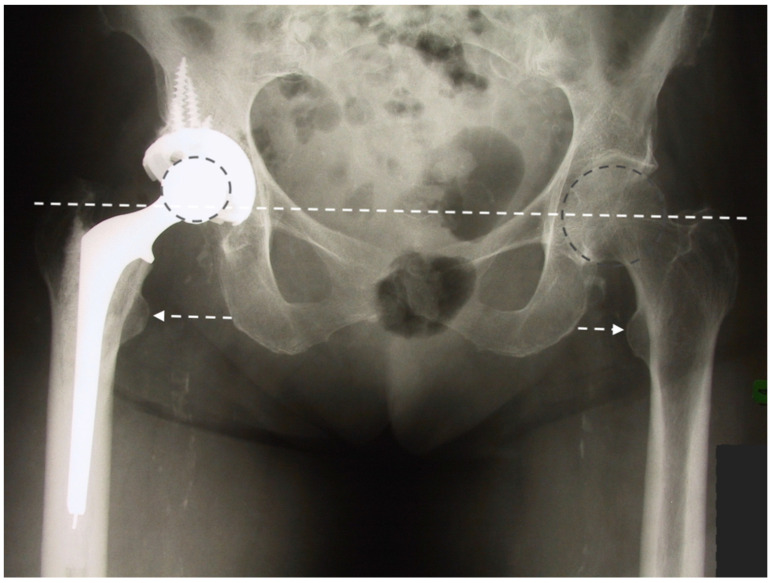
Anteroposterior pelvis radiograph of a patient complaining of right hip pain. Compared to his native left hip, the right 28 mm THA center of rotation was elevated, the femoral offset was increased, and leg length was shortened.

Precise biomechanical reconstruction of the hip by THA is essential for the success of this procedure. Optimal femoral offset and leg length restoration result in better abductor strength and clinical function. Failure to restore the normal anatomy for THA has been associated with a higher rate of dislocation, muscle weakness, limping, leg-length discrepancy, impingement, and early loosening of the implant (Figure 4) [12]. To improve the precision of the anatomic reconstruction, the range of sizes of the implant has been increased. Modular options have been introduced to give different neck-shaft angles and offsets of the stem. To enable the surgeon to achieve optimal restoration of the hip joint anatomy, different implant modularities, geometries, sizes, and computer navigation systems are now available.

**Figure 5:**
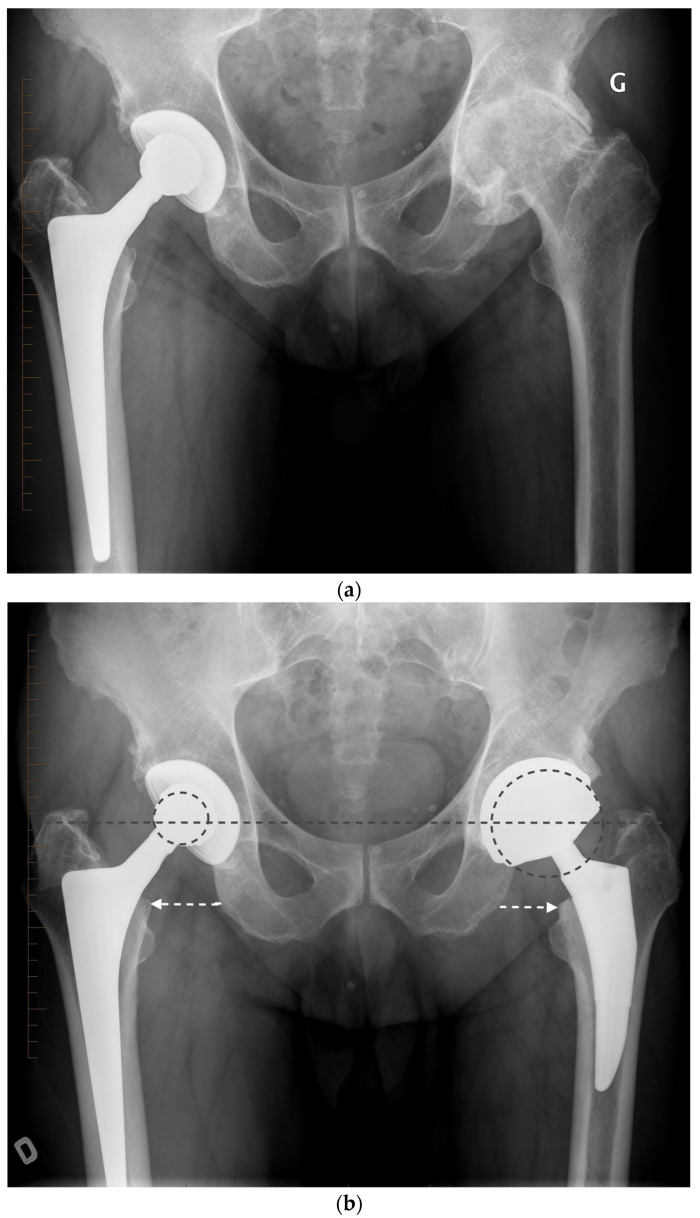
(**a**) Antero-posterior pelvis radiograph of a 55-year-old man with a right metal-on-metal 28 mm THA and severe osteoarthritis of the left hip. (**b**) Postoperative radiograph after a left LDH THA with leg length and femoral offset restoration.

We prefer to use a fixed neck stem with three different neck angle options to better meet the different patients’ anatomies rather than a stem design with a unique angle and two different offsets (standard/lateralized). Less influenced by the risk of instability, surgeons using LDH THA can better optimize a patient’s leg length and femoral offset (Figure 5). In our experience and the literature, up to 10 mm of leg length difference can be tolerated; a shorter leg is much better tolerated than a longer one by patients [15]. In the cases where we hesitate between a shorter or a longer head (example: 0 mm vs. a +4 mm), we, therefore, select the shorter one.

#### 3.2.2. Goal #2 of a Personalized THA: Minimize Abnormal Stress Transfer to Bone 

Femoral stem designs have specific load patterns. These load patterns do not replicate the physiological load pattern exerted on the proximal femur, instead they result in an adaptive periprosthetic bone remodeling. The location and magnitude of stress transfer from the femoral stem to the bone vary depending on mechanical and biological factors; the former is related to the implant, and the latter is associated with the stiffness of the patient’s bone. In younger and more active patients selecting a femoral implant with lower implant-related thigh pain is of primary importance to allow these patients to return to impact sports or demanding work. Our preferred stem is an uncemented titanium collarless tapered stem that obtains a press-fit in the metaphysis and the metaphyso-diaphyseal junction. The tapered shape prevents a complete fill in the distal diaphyseal portion, encouraging a more physiological load transfer in the proximal part of the femur. In a randomized control trial (RCT) comparing hip resurfacing (HR) to THA (with the CLS implant, Zimmer-Biomet, Warsaw, USA), patients reported occasional thigh pain related to activity in 6% of THA and 2% of HR after a 6–9 years follow-up [16]. 

For the older, less active patients, and often with osteopenia, we favour tapered, polished, cemented stems. This strategy promotes excellent clinical results for this patient group and reduces the risk of periprosthetic fracture (intra and postoperative). In addition, the Australia Orthopaedic Association National Joint Replacement Registry states, that for the over-75 age category, there is a lower revision rate for cemented and hybrid implants when compared with uncemented [17].

In the long term, bone remodeling around a stem is an unavoidable physiological process related to implant design. However, for some predisposed patients, it can lead to periprosthetic bone loss secondary to severe stress-shielding, which is thought to contribute to late loosening, late periprosthetic fracture, and, thus, renders revision surgery more complicated. Interestingly, despite all the differences between the various stem designs, a review of the best-performing cementless stems found no evidence that one design is superior to another in terms of clinical outcome or extended long-term survival [18].

#### 3.2.3. Goal #3 of a Personalized THA: Offer Unrestricted Hip Range of Motion and Stability

(3a) Avoid Hip Instability

**Figure 6:**
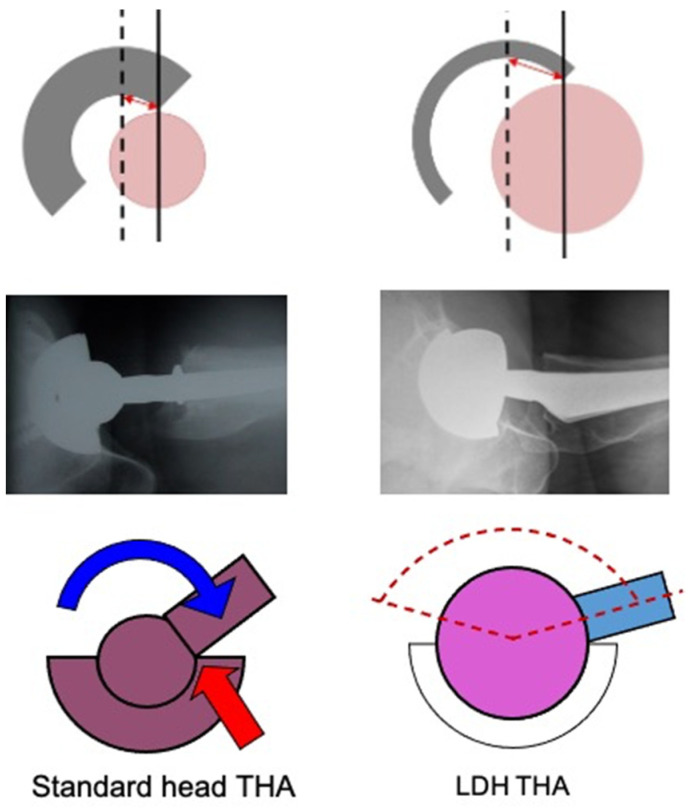
Composite image showing the differences in the jump distance, femoral head-neck offset, and impingement free range of motion between a standard femoral head and LDH.

Dislocation is one of the leading causes of THA revision with a variable incidence in the literature. Determining the precise incidence of dislocations is complex as closed reductions might remain undetected. Hermansen et al. sought to find a true cumulative incidence of dislocation in 30,000 THAs in the Danish registry and reported a rate of 3.5% [19]. The primary arguments for LDH implantation are a wider impingement-free range of motion and a reduced risk of dislocation due to increased jump distance and the larger head size to displace (Figure 6). Zijlstra et al. analyzed 160,000 THAs from the Dutch arthroplasty register and found that the cumulative risk of dislocation was significantly higher in 22–28 mm heads (1.1%), compared to >36 mm heads (0.5%) [20,21,22]. In our practice, with more than 3500 CoC LDH THAs implanted since 2011, we encountered 5 (0.14%) early postoperative dislocations, of which four were successfully treated with closed reduction, and one had recurrent dislocations that required revision surgery. Once instability is of lesser concern, leg length adjustment and femoral offset restoration can be performed with more ease (which promote achievement of goal #1). Using intraoperative measurements or computer/robotic assistance, the surgeon can focus on anatomic restoration without the fear of instability. Knowing that patients would better tolerate a shortened leg to an elongated one, we favour the shorter one when in doubt between two head lengths [15].

**Figure 7:**
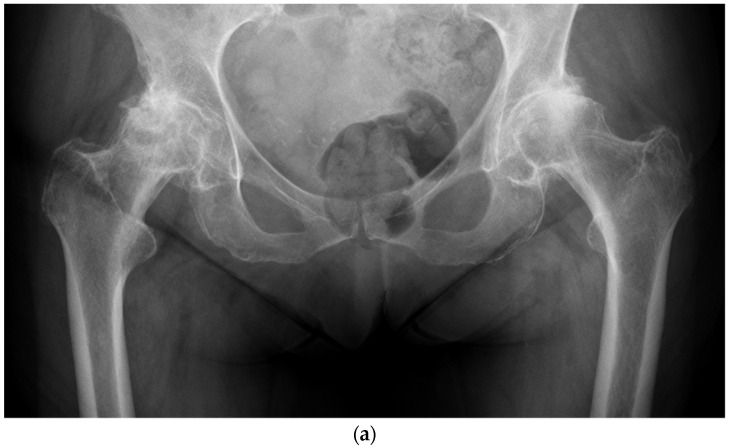

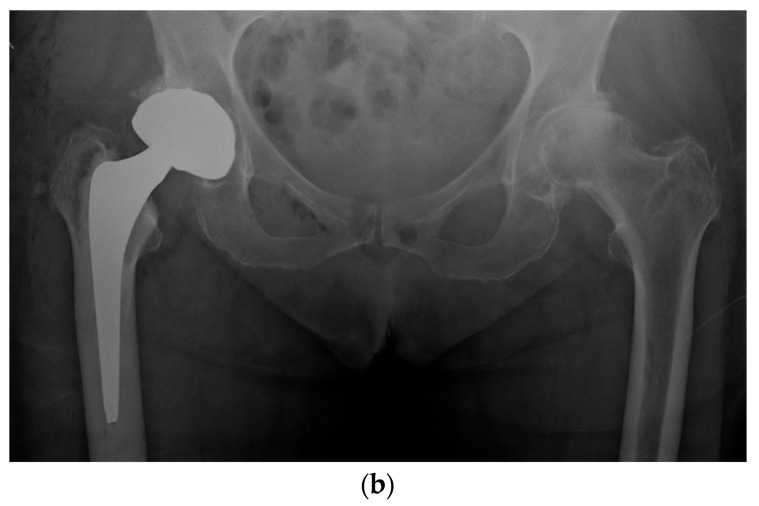
(**a**) Antero-posterior pelvis radiograph of a 75-year-old woman with severe bilateral hip osteoarthritis. During the right THA surgery, the acetabular cavity was reamed to 47 mm. A monobloc acetabular component of 48 mm was implanted with a DM polyethylene head of 41 mm (28 mm metal head). A polished tapered stem was cemented. (**b**) Post-operative anteroposterior pelvis radiograph.

Using a LDH THA allows unrestricted movement after THA for all types of surgical approaches. Vendittoli’s group in Canada practices no postoperative ROM restriction for the posterior surgical approach, as it simplifies patients’ education, enhances their confidence during rehabilitation, and facilitates bilateral procedures [23,24]. LDH monobloc DM is especially interesting for many older women with large femurs and a small acetabular cavity (<50 mm, Figure 7). In these cases, using a standard bearing diameter is prone to instability. With its LDH, DM design provides optimal implant stability for these not so infrequent patients. Moreover, with LDH, we do not impose activity restrictions in the long term. LDH CoC THA offers a significant benefit for many active individuals who can return to their regular jobs and for those who want to practice demanding sporting activities.

(3b) Optimize Hip Micro Stability

The LDH is closer to the native femoral head size. It can be speculated that reproducing a more anatomical joint with more natural capsular tension and proprioception can produce better results. It has also been shown that LDH generates suction forces up to 30 N (3 kg) between the femoral head and acetabular liner and reduces implant micro-separation [23,25]. The larger the bearing diameter and smaller the component clearance, the higher is the suction force. This phenomenon is particularly important with CoC designs, as they allow higher surface wettability and, consequently, higher cohesiveness of the lubricating film than implants with PE surface [23].

(3c) Allow Unrestricted Hip Range of Motion

Given the constant neck diameter for most femoral stem designs, the head–neck ratio increases proportionally by increasing the head diameter. This leads to a theoretical and clinical greater hip range of motion amplitude [26,27]. In addition, owing to a supra physiologic arc of motion, extra-articular impingement occurs first, and inter-component impingement is rare. Consequently, less than optimal implant positioning can be better tolerated [28]. This is an essential notion considering the extent of surgical indications for patients with primary or secondary anatomy deformations (e.g., dysplasia, acetabular retroversion, previous pelvic osteotomy, post-traumatic, etc.) as optimal implant positioning is often challenging (Figure 7). 

LDH Bearing Is a Forgiving Implant

The interrelation between the spine, pelvis, and hip was recently recognized as a critical factor explaining component impingement, limited ROM, and instability after THA with standard bearing diameters [29,30,31,32,33,34]. Undoubtedly there is a benefit in considering static and dynamic spinopelvic parameters [35], which will become an integral part of preoperative planning. However, despite the scientific interest, it has somewhat limited use in daily practice. Applying the recommendations for a functional implant alignment requires sophisticated preoperative 3D imaging techniques and intraoperative precision tools to apply the preoperative plan (precise acetabular component orientation ±2–5 degrees). Being a forgiving implant (mal-alignment of LDH is less likely to lead to a dislocation due to the jump distance), LDH THA is a much simpler solution. The supraphysiologic ROM offered by the large head-neck offset can compensate for patients’ abnormal spinopelvic mobility and surgeons’ imprecision.

Furthermore, over a lifetime, spinopelvic mobility and parameters might change; LDH THA should sustain these unpredictable modifications. When implanting LDH acetabular components, our goal is to obtain a functional alignment of the bearing (congruency between the femoral and the acetabular components) with the hip in 10–20° of flexion, 10° of adduction and 20–30° of internal rotation. This is the modified Ranawat’s sign published by Thomas J. Blumenfeld [36]. 

#### 3.2.4. Goal #4 of a Personalized THA: Provide a Forgotten Joint

A double-blind RCT was performed comparing HR to metal-on-metal (MoM) LDH THA with a control group of patients without a hip replacement. Patient gait speed, postural balance, and performance on several functional tests were evaluated in a gait laboratory [37]. The performances at most functional tests and clinical scores were similar between HR, LDH, and the control group at three months postoperatively. By these measures, HR did not provide better clinical function over LDH THA, and both restored a normal hip performance by three months post-surgery. Using a CoC LDH THA in 276 patients, we reported an excellent mean WOMAC score of 92.3, UCLA activity score of 6.6, and Forgotten Joint Score of 88.5 after a mean follow-up of 67 months [24]. 

As a patient’s perception of a normal joint is the ultimate THA goal, we previously described and validated a simple question with five possible answers (Patient’s Joint Perception question: PJP) [38]. Using this simple question, we published that 52% of LDH THA patients had a “natural joint” perception, and 24% of patients reported no limitations after their LDH THA [39]. These clinical results show that modern hip replacements have fulfilled the aim of achieving a forgotten hip for the majority of patients.

#### 3.2.5. Goal #5 of a Personalized THA: Lifetime Implant Survivorship

THA implant survivorship should exceed the patient’s life expectancy. Polyethylene wear-related biological reactions in young and active patients continue to be a major concern. Orthopaedic surgeons have attempted to address this issue by using alternative hard-on-hard bearings. In comparison to metal-on-polyethylene (MoP) bearing, CoC offers greater scratch resistance and lower wear debris production [40]; it was also linked to a reduction in wear-induced osteolysis, reduced cumulative long-term risk of dislocation, reduced corrosion of the head-neck modular junction, and lower revision rates. The evidence in the UK National Joint Registry 2021 states that the lowest revision rates are for uncemented CoC bearings compared with the highest revision rates for MoP for head sizes above 36 mm [41]. In a recent published RCT, we compared the long-term implant aseptic revision rate. Mean follow-up of 21 years, of MoP (conventional poly, 17/69; 24.6%) vs. CoC (2/71; 2.8%; *p* < 0.001) [42]. Kaplan–Meier survivorship analysis for aseptic revisions was 73.6% (95% CI: 63.3–84.9%) for MoP while it was 96.9% (95% CI: 92.8–100%) for CoC (*p* < 0.001). Moreover, 61% (14/23) MoP vs. 6% (2/33) CoC showed osteolytic signs (*p* < 0.001). The evidence in the literature suggest that thinner PE inserts have a faster wear rate. Historically, minimum PE thickness has been 8 mm to prevent faster wear and also prevent catastrophic failure [43]. Our findings align with a systematic review of RCTs and meta-analysis comparing THA with six different bearings with more than ten years of follow-up [42]. The authors reported a higher risk of revision of MoP (conventional) compared to CoC (RR: 2.83; 95% CI: 1.20–6.63). A meta-analysis including 5 RCTs (n = 974 hips; 601 CoC and 373 MoPc) found lower osteolysis and fewer radiolucent lines with CoC (4.4% vs. 18.1%, respectively; RR = 0.22; 95% CI: 0.14–0.36; *p* < 0.01) [42,44]. This wear-resistant material makes it the ideal option for a LDH bearing. We reported the results of our first consecutive 264 CoC LDH THAs after a mean follow up of 67 months (48 to 79) postoperatively [24]. There were four re-operations (1.5%), including one early revision for failure of acetabular component primary fixation (0.4%). No hip dislocation was reported. For the younger, active patient with a life expectancy of more than 20 years, LDH CoC is a sound option.

**Figure 8:**
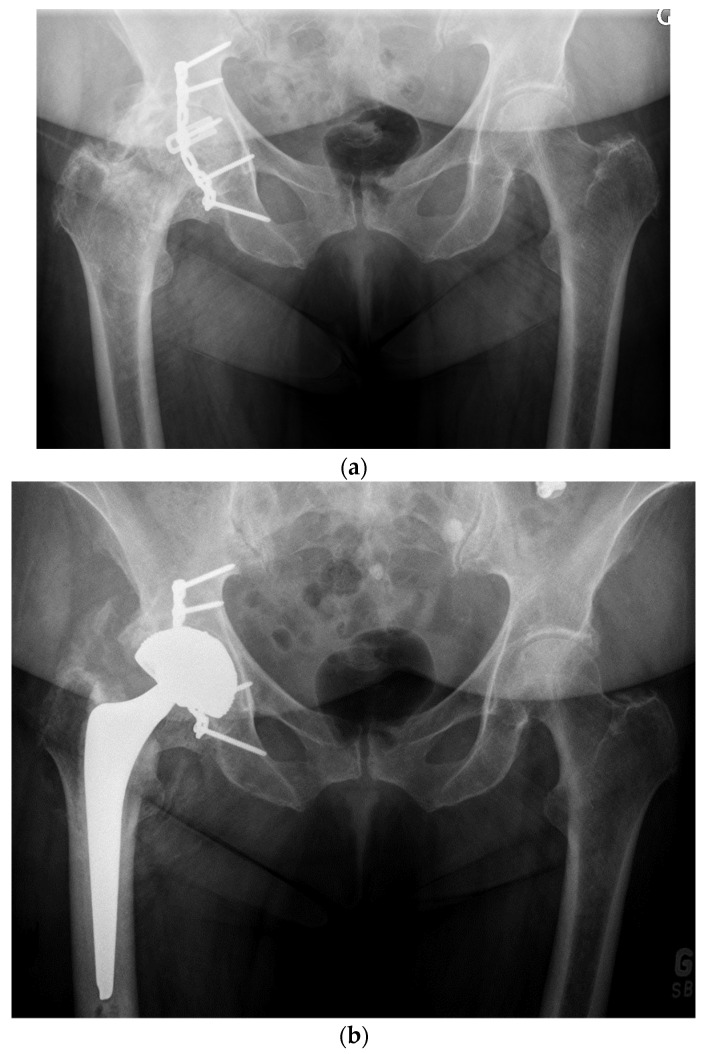

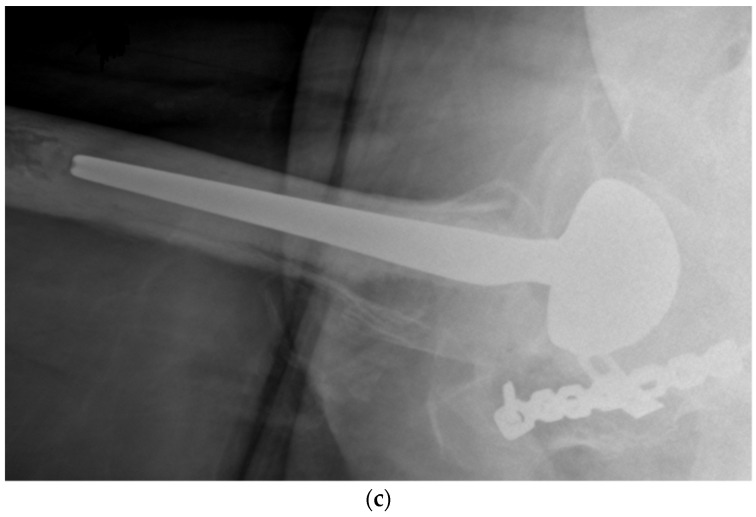
(**a**) Antero-posterior pelvis radiograph of an 82-year-old man with severe post-traumatic right hip osteoarthritis. The fracture and secondary degeneration modified native acetabular cavity orientation. Using a DM, LDH THA helped obtain a stable joint and may forgive potential surgical impressions. (**b**) Post-operative anteroposterior pelvis radiograph. (**c**) Lateral view of the reconstructed right hip.

For the older, more sedentary patient or the patient with a shorter life expectancy, DM LDH THA is the ideal alternative (Figure 7 and Figure 8). DM implant design has the LDH stability benefits and wear rates significantly lower than fixed bearing implants [45,46]. Loving et al., in a simulator study, under multiple test conditions (impingement, abrasion, loss of mobility of the insert), showed that the performance in terms of wear was dictated mainly by the smaller articulation and by the polyethylene material used. For the most severe tests, a 75% lower wear rate was observed compared to a fixed insert of conventional polyethylene sterilized under gamma rays in an inert atmosphere [47]. Additionally, with osteopenic bone there is a higher risk of intra-operative periprosthetic fractures with LDH press fit monoblocks [48,49]. Bearing a lower cost and avoiding the potential drawbacks of hard-on-hard bearings (noise, fracture, and trunnionosis), DM should be considered for patients over 65 years.

## 4. LDH THA Potential Downsides

### 4.1. Volumetric Wear

LDH are not without potential complications. Traditionally, the volumetric wear associated with larger diameter heads was a common subject of discussion. The volumetric wear is an inherent trait of PE liners, and its minimal thickness has been proposed to be 8 mm. With increased quality and wear resistance of the PE, these values were reduced to 6 mm or even to 3.9 mm for ultra-high molecular weight PE [50]. Despite the high resistance of new generation PE, the head diameters are still limited, and the long-term bearing wear for a thinner PE is still unknown. At present, we prefer to use CoC or DM bearings as both bearings have been shown to produce minimal wear with LDH [46]. 

### 4.2. Trunnionosis

Taper wear and corrosion, also known as trunnionosis, have been reported in the literature [51]. The galvanic corrosion was observed less often when the head–neck junction combination was from the same material. For example, CoCr/CoCr is less susceptible to corrosion than CoCr/Ti or CoCr/Stainless steel couples. Many factors, such as high offset, low neck/shaft angle, taper geometry (length, angle), surface finish, and even the angle and the force of impaction of the head were investigated and attributed to the risk of corrosion [51]. Panagiotidou et al., in their retrieval study, demonstrated that using ceramic heads significantly reduces the fretting and corrosion when used either on Ti or CoCr stems [52]. 

We reported the Ti blood concentration in 57 CoC LDH THAs with Ti stem. We showed that the Ti ion levels were normal and stable at two years (2.2 µg/L) and five years postoperatively (2.0 µg/L, statistically significant reduction, *p* = 0.007) [53]. Moreover, we did not find a correlation between Ti levels and femoral head size (40, 44, and 48 mm). In addition, the use of Ti sleeve did not show a significant difference in blood Ti ion concentration. With regard to DM, using a small 28 mm head on the stem trunnion retains the benefits of LDH while minimizing the risks of trunnionosis. Such a head–trunnion combination has been used with success for decades.

### 4.3. Ceramic Liner Fracture 

Ceramic liner fractures mostly occur at liner insertion or after inappropriate surgeon impaction (malseated liner). Pre-assembled ceramic liner in LDH CoC acetabular component virtually eliminated these risks. With more than 3500 LDH delta ceramic cases, we had no liner or head fracture case. 

### 4.4. Ceramic Articular Noises

Our group reported a rate of at least one episode of audible noise in 22.7% of LDH CoC THAs, which was strongly associated with young and active patients and with a larger femoral head diameter [24]. Though the audible noises might be distressing for the patient, they are mostly occasional and benign, and not associated with a reduced functional score or patient dissatisfaction. Chatelet et al. similarly reported 28% of patients with CoC THAs had some articular noise, but its effect on daily quality of life was negligible and the overall survival of was 94.2% at 11 years follow-up. [54]. 

## 5. Conclusions

In our experience, LDH THA with either CoC or DM configuration has proven to be very efficient in achieving most goals of personalized hip arthroplasty. The large head-neck ratio of LDH THA almost removes the risk of dislocation, creates a supraphysiologic arc of motion, and allows a certain degree of surgical imprecision in acetabular orientation. Hence, it is a forgiving procedure, even in patients with impaired spinopelvic kinematics. Less impacted by the risk of instability, LDH also allows a better restoration of individual hip anatomy regarding capsular tension, femoral offset, and leg length—thus favouring pre-disease hip kinematics.

In addition, LDH eliminates postoperative restrictions, helping rehabilitation and speeding up the return to daily, occupational and sports activities. To provide an implant for life, ceramic is the ideal bearing for young and active patients with a life expectancy >20 years. For the other patient cohorts, DM is an attractive alternative due to its lower cost. Overall, LDH THA, either CoC or DM improves patient satisfaction, clinical outcomes and is an excellent option on our path to offer a personalized and forgotten hip joint.
